# Solomon Islands Largest Hawksbill Turtle Rookery Shows Signs of Recovery after 150 Years of Excessive Exploitation

**DOI:** 10.1371/journal.pone.0121435

**Published:** 2015-04-08

**Authors:** Richard J. Hamilton, Tomas Bird, Collin Gereniu, John Pita, Peter C. Ramohia, Richard Walter, Clara Goerlich, Colin Limpus

**Affiliations:** 1 The Nature Conservancy, Asia Pacific Division, 245 Riverside Drive, West End, Brisbane, QLD, 4101, Australia; 2 ARC Centre of Excellence for Coral Reef Studies, James Cook University, Townsville, QLD, 4811, Australia; 3 School of Geography and Environment, University of Southampton, Southampton, SO17 1BJ, United Kingdom; 4 The Nature Conservancy, Solomon Islands Field Office, PO Box 759, Honiara, Solomon Islands; 5 The Nature Conservancy, Isabel Environmental Office, Buala, Isabel Province, Solomon Islands; 6 ADB Coral Triangle of the Pacific Project, Ministry of Environment, Climate Change, Disaster Management & Meteorology, Honiara, Solomon Islands; 7 Department of Anthropology and Archaeology, University of Otago, Dunedin, New Zealand; 8 Universität Würzburg, Würzburg, Germany; 9 Threatened Species Unit, Department of Environment and Heritage Protection, Queensland Government, 41 Boggo Rd., Dutton Park, Brisbane, QLD, 4102, Australia; University of Tennessee, UNITED STATES

## Abstract

The largest rookery for hawksbill turtles in the oceanic South Pacific is the Arnavon Islands, which are located in the Manning Strait between Isabel and Choiseul Province, Solomon Islands. The history of this rookery is one of overexploitation, conflict and violence. Throughout the 1800s Roviana headhunters from New Georgia repeatedly raided the Manning Strait to collect hawksbill shell which they traded with European whalers. By the 1970s the Arnavons hawksbill population was in severe decline and the national government intervened, declaring the Arnavons a sanctuary in 1976. But this government led initiative was short lived, with traditional owners burning down the government infrastructure and resuming intensive harvesting in 1982. In 1991 routine beach monitoring and turtle tagging commenced at the Arnavons along with extensive community consultations regarding the islands’ future, and in 1995 the Arnavon Community Marine Conservation Area (ACMCA) was established. Around the same time national legislation banning the sale of all turtle products was passed. This paper represents the first analysis of data from 4536 beach surveys and 845 individual turtle tagging histories obtained from the Arnavons between 1991-2012. Our results and the results of others, reveal that many of the hawksbill turtles that nest at the ACMCA forage in distant Australian waters, and that nesting on the Arnavons occurs throughout the year with peak nesting activity coinciding with the austral winter. Our results also provide the first known evidence of recovery for a western pacific hawksbill rookery, with the number of nests laid at the ACMCA and the remigration rates of turtles doubling since the establishment of the ACMCA in 1995. The Arnavons case study provides an example of how changes in policy, inclusive community-based management and long term commitment can turn the tide for one of the most charismatic and endangered species on our planet.

## Introduction

Sea turtles are considered flagship species for conservation and their plight has captured widespread attention. Worldwide declines in sea turtle populations have primarily been attributed to excessive exploitation with global catches peaking at over 17,000 tonnes in the late 1960s [1]. In the past four decades changes in policy have resulted in greater protection of marine turtles, particularly through global restrictions on the trade of turtle products under the Convention on International Trade in Endangered Species of Wild Fauna and Flora (CITES) [1]. Yet, turtles remain under threat from climate change [2, 3], illegal trade [4], by-catch [5] and legal subsistence take [1].

In the Pacific there is archaeological evidence of turtle hunting leading to cases of extirpation and population decline going back at least 3000 years [6]. The effect of prehistoric Pacific turtle hunting was ecologically complex. For example, Allen [6] shows that turtle harvesting in the colonisation phase of West Polynesia (~900 BC) may have impacted on turtle populations thousands of kilometres away in East Polynesia long before those islands were settled by humans. In Solomon Islands the critically endangered hawksbill turtle (*Eretmochelys imbricata*) has formed an important component of the cultural value systems and subsistence economies for centuries [6, 7], and it played a central role in early commercial exchange with Europeans. Trade between Europeans and islanders from New Georgia archipelago in the Western Province of Solomon Islands started in the first decades of the nineteenth century and by 1840 by far the most important trade item was the shell of hawksbill turtles [8, 9].

By the mid nineteenth century certain chiefs from Roviana Lagoon, New Georgia dominated this trade and were able to amass enormous power [10]. In return for turtle shell the Roviana chiefs sought iron, and would trade for little else [8]. The Roviana Chiefs practiced headhunting and iron was sought to fashion tomahawks that were used in the headhunting raids. At first the Roviana traders targeted hawksbill turtles from their inshore reefs and lagoons. But the absence of large hawksbill rookeries in New Georgia archipelago [11] quickly incentivised headhunting parties to raid into Isabel and Choiseul, where the real target was the nesting sites in Manning Strait, especially the Arnavon Islands [12] which support the largest rookery for hawksbill turtles in the oceanic South Pacific [13]. Turtle shell trading and raiding for slaves and heads formed an interlocking system that led to increasing levels of violence in the Western Solomon Islands [14]. A few astute chiefs who had already monopolised the turtle trade at the point of sale, were able to gain direct control over the most lucrative point of supply though the mobilisation of armed raiders. From 1840–1900 thousands of hawksbill turtles were killed each year in Solomon Islands for trading purposes [9].

By the twentieth century human (and presumably hawksbill) populations on Choiseul and Isabel had been depopulated by Roviana headhunters [9], and in 1904 the seemingly unoccupied and uncultivated Arnavon Islands and surrounding islands in the Manning Strait were declared alienated land under the Waste Lands Regulations Act (1904), becoming government property in a section of public lands known as the Wagina block [7, 9]. In that same year Captain William Hamilton received a 99 year lease to the Wagina Block and rights to harvest turtle shell and pearls from this region [9]. Legal ownership of the Arnavons was transferred back to the national government in the 1930s and the islands became open access, with an abundance of trochus, crocodiles and turtles continuing to attract traders to the area [7]. In 1963 the British government resettled people from the Phoenix Islands in Ikiribati to Wagina Island and these Micronesian people also began harvesting turtles from the Arnavons [11].

After WWII all hawksbill turtle shell coming out of Solomon Islands was exported to Japan [4], and by the mid-1970s it had become apparent to the Ministry of Natural Resources (MNR) that the Arnavons were a highly significant nesting area for hawksbill turtles and that its population was in rapid decline [11]. In recognition of this the MNR designated the Arnavons as “off limits” under a trespass law, with the intent of creating a turtle sanctuary [7]. A turtle monitoring, rearing and enforcement project commenced at the Arnavons in 1976 [11]. While these early conservation efforts were commendable, the approach was heavy handed and it failed to engage the traditional owners of the Arnavons. Consequently in 1982 Rence Zama of the Volekana tribe burned down infrastructure that had been established on the Arnavons, an act he publicly announced (Rence Zama, personal communications). He was imprisoned for his efforts but the turtle project at the Arnavons ceased and turtle hunting resumed. In the following decade hunting for hawksbill turtles intensified due to strong demand for hawksbill shell from Japan [15]. In the late 1980s exports from Solomon Islands peaked with over 4000 adult hawksbills being killed each year to supply Japanese markets [4]. In late 1993 amendments to the Solomon Islands Fisheries regulations banned the sale, purchase and export of any turtle product, which effectively saw large scale trade in hawksbill turtle shell cease [16]. Under the 1998 Fisheries Act it is now also illegal to take turtle eggs or destroy their nests during the breeding seasons of June to August and November to January. Subsistence take of turtles is still permitted, with resting hawksbills frequently captured at night by free diving fishers’ who use fins, mask, snorkel and an underwater flashlight to search shallow reef slopes [17, 18].

In 1991 The Nature Conservancy (TNC) entered Solomon Islands and began to work with local resource owners and the Choiseul and Isabel provincial governments to re-establish the Arnavons sanctuary, this time with a strong focus on community-involvement and education [19–21]. Routine beach surveys and tagging of nesting hawksbills commenced in 1991, along with extensive consultations with the communities of Kia, Katupika and Wagina regarding the future of the Arnavons [22, 23]. In 1995 the Arnavon Community Marine Conservation Area (ACMCA) was formally established. The ACMCA protects 152 km^2^ of land and sea; the first and largest community based protected area in Solomon Islands. The ACMCA is supported by Isabel Province legislation and has a management committee made up of representatives from the communities of Kia, Wagina, Katupika, TNC, The Ministry of Environment, Climate Change, Disaster Management and Meteorology (MECDM) and Isabel and Choiseul provincial governments. Since 1995 conservation officers from Kia, Wagina and Katupika have been based on the Arnavons year round. Conservation officers’ carry out the turtle monitoring programs and enforce the ACMCA management measures. Despite the constant presence of the conservation officers varying levels of poaching (0–10 incidents per year) are known to have occurred at the Arnavons since 1995.

In 2006 TNC established an endowment that currently covers a third of the costs of running the Arnavons and in 2011 all assets and management responsibilities were transferred from TNC to the ACMCA committee. Over the past decade ecotourism has also steadily grown at the Arnavons (www.arnavons.com), and in 2008 the ACMCA won the United Nations Equator Prize, which is awarded biennially to recognize outstanding community efforts to reduce poverty through the conservation and sustainable use of biodiversity. Yet despite being upheld as a conservation success story the Arnavons 22 year (1991–2012) turtle monitoring database has never been analysed. The aim of the present study is to draw on the monitoring data in order to understand the population dynamics of ACMCA hawksbill turtles—with particular attention to migration, annual nesting rates and remigration rates. An evaluation of these parameters will be used to assess the efficacy of the conservation initiative, and to provide insights on the effects of different episodes of high and low human predation.

## Methods

### Ethics statement

The study adhered to the legal requirements of Solomon Islands. All fieldwork was carried out with approval from the Ministry of Agriculture and Fisheries and the MNR (now known as MECDM), Honiara, Solomon Islands. Access to the study area for research purposes was permitted under the 1995 ACMCA management plan.

### Study area

**Fig 1:**
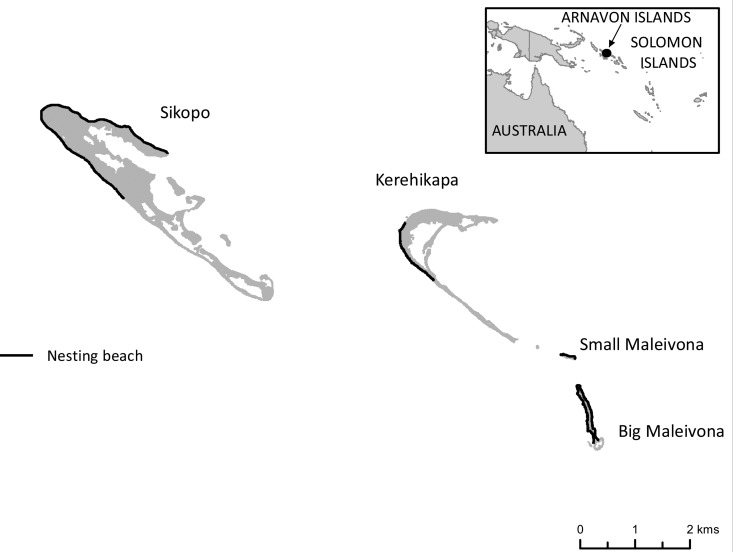
The four main islands that make up the Arnavons and their nesting beaches. The location of the Arnavons group is shown in the insert.

The Arnavons are made up four small islands located in the Manning Strait between Choiseul and Isabel Province (Fig. 1). The largest island is Sikopo, which has a nesting beach that is 5042 m long. The second largest island is Kerehikapa and it has a nesting beach that is 1484 m long. The field station is based on Kerehikapa. Big Maleivona has a nesting beach that is 2544 m long and Small Maleivona has a nesting beach that is 346 m long (Fig. 1). The basic data utilised in this study derived from three linked survey programmes; daily nest counts and night flipper-tagging surveys at Kerehikapa and weekly nest counts at the three other islands.

#### Daily turtle nest counts – Kerehikapa

Between May 1991 and June 2012 3680 daily turtle nest counts were conducted along the entire length of Kerehikapa nesting beach (S1 Table). From 1991–1995 and in 2000 beach surveys were conducted before noon only between the months of May and September (S1 Table). Successfully laid nests were given an individual nest number and moved above the high tide mark if required. In 2001 the Arnavons turtle monitoring program was reviewed [13]. Based on this review, conservation officers began conducting daily surveys of Kerehikapa beach throughout the year from late 2001 onwards, using the same protocols described above (S1 Table). Data from surveys conducted between 1996–1999 were destroyed when the TNC office in Honiara burnt down in 2005.

#### Weekly beach surveys

Weekly beach surveys were conducted along the entire nesting beaches at Sikopo, Big Maleivona and Small Maleivona. In total 856 weekly beach surveys were conducted across these three islands between May 1991 and October 2012 (S2 Table). Between 1991–1995 and in 2000 surveys were conducted once a week during the months of May—September (S2 Table). To prevent the possibility of double counting a nest on subsequent surveys, all successfully laid nests were clearly marked. At all beaches conservation officers recorded “Pits Only” (when a nesting pit was visible but not the tracks), and “Pits and Tracks” (both pit and tracks visible). Following the program review in 2001 ACMCA conservation officers conducted weekly monitoring throughout the entire year. Logistical constraints meant that from 2001–2012 monitoring occurred approximately fortnightly (S2 Table).

#### Flipper tagging of nesting turtles

Tagging of nesting hawksbills took place during night time hours over the 22 year sampling period (1991–2012). All tagging was conducted at the Kerehikapa nesting beach. From 1991–1995 tagging surveys were conducted between the months of May—September [19, 20, 23], the peak nesting period for the Arnavons group [7, 11]. Nightly beach surveys were conducted from sunset until midnight, with nesting turtles double tagged at first encounter using uniquely numbered titanium tags [24]. From 1996 onwards trained ACMCA conservation officers conducted tagging throughout the entire year. Our data set comprised the capture mark recapture histories for 845 individual nesting female hawksbills sampled over the 22 year period.

### Modelling population dynamics

In this study we focus on three population parameters; migration (where the turtles went when they were not in the Arnavons), yearly estimates of laying activity (numbers of nests laid per night and changes in this value over time) and remigration rates (the percentage of mature females successfully returning to nest in a subsequent breeding season). We discuss the methodologies used in these three studies below.

#### Migration

To examine the migration pattern of turtles that use Kerehikapa beach we reviewed existing data on two tag recoveries [25, 26] and information on two nesting females that were tagged with satellite transmitters at Kerehikapa in July and August 2001 [13]. In the process of cross checking the 1991–2012 Kerehikapa database we also discovered one hawksbill turtle that was initially tagged as a juvenile in the northern Great Barrier Reef (GBR), Australia [27].

#### Yearly estimates of laying activity

Prior to 2001, data for each beach were collated in data records as the total number of nests laid (pits and tracks and pits only) during the peak laying season (May to September). These totals were divided by the survey duration (on the order of 66–90 days) in each year to get a mean number of nests laid per night for that year [19, 20, 23, 28]. From 2001 onwards we used counts of pits and tracks for our analysis. Because tracks can rapidly be washed out on high tides or covered by sand, weekly counts of pits and tracks likely provide an underestimate (relative to pre-2001 methods) of nesting activity. Because of the varying levels of effort over time, we standardized beach count data as follows. We first calculated the average number of nests seen on each weekly survey conducted during the peak laying period (May to August). To make these counts comparable with pre-2001 counts, we divided the average number of nests seen on weekly surveys by seven, thereby producing an estimate of the mean number of nests laid per night. Given that from 2001 onwards weekly counts of pits and tracks tended to occur less than four times a month (S2 Table), the 2001–2012 estimates for Big Maleivona, Sikopo and Small Maleivona can be considered to be conservative. From 2001 Kerehikapa surveys continued to be done intensively (S1 Table), so we divided the number of nests seen within a month by the number of surveys performed in that month. To examine the seasonality of nesting we plotted the average number of nests laid per night in each month for the years 2001–2012.

To test for a significant trend in nest numbers over time we used a linear regression model of the mean number of nests observed per night per beach against years since 1991 (i.e. # nests ~ f (beach + year + beach × year), using the software R [29]. We also tested three other nested models that included only year as a covariate, beach only and year + beach (without the interaction). We examined the relative goodness of fit for each of these four models using Aikeke’s information criterion (AIC) and report the results from the best-fit model. We also used a t-test to test for a difference between the average number of nests laid per night in the entire Arnavons group in the pre protection period (1991–1995) and in the most recent post protection period (2008–2012).

#### Remigration rates and remigration intervals

While in principle our tagging data could be analysed in a capture mark recapture (CMR) model to estimate annual survival rates and remigration intervals, standard CMR analyses provided significantly biased estimates of survival and capture rates. In simulation studies, we found that the turtle’s nesting cycle is a case of non-random temporary migration, posing a significant challenge to performing standard CMR analyses ([30], and see further details in document S3 Text. Analytical considerations with the CMR data). Instead, we used the proportion of turtles from a cohort that were ever resighted as an indicator of overall survival. In order to determine if the proportion of the population remigrating increased following the establishment of the ACMCA we calculated the proportion of each cohort in the pre protection era (1991–1995) that remigrated. Because pre-protection cohorts had a greater time period in which they could have returned (i.e. between 1991 and 2012 = 21 years), there was a potential bias in this proportion. We therefore restricted our calculations to within a 12 year window of each year, a timeframe that encapsulates the remigration period for most turtles.

For the purposes of this analysis, we defined a cohort as being comprised of all turtles observed nesting in a particular year, regardless of whether they had been observed previously. For each year, we calculated what percentage of that cohort was ever recaptured in the 12 years following 1991 (i.e. to 2003). We did the same for each year up to 2000 as this was the latest year for which we could use the 12 year window. We then calculated an average remigration rate across all years in the pre protection (1991–1995) and post protection (1996–2000) cohorts and used a t-test to determine whether there was a significant difference between the two.

We also calculated the mean remigration interval, or frequency with which turtles returned to Kerehikapa beach, for the pre and post protection periods. In each yearly cohort we looked at all turtles that were captured within a year and calculated the time to their next capture (if at all). We then took the average over all turtles within that year that were ever resighted. Once a turtle was resighted, it was treated as a member of the cohort for that year. So if a turtle was seen in 1991, 1999 and 2003, we calculated two remigration intervals of 8 years and 4 years respectively, and it was treated as being a member of the 1991, 1999 and 2003 cohorts. We then tested for a significant decline in the mean remigration interval for the cohorts between 1991 and 2001 using a poisson regression of yearly mean remigration interval against year, setting the maximum remigration interval to 12 years to avoid a bias against longer remigration intervals in later cohorts.

## Results

The results of the three studies are outlined below.

### Migration

**Fig 2:**
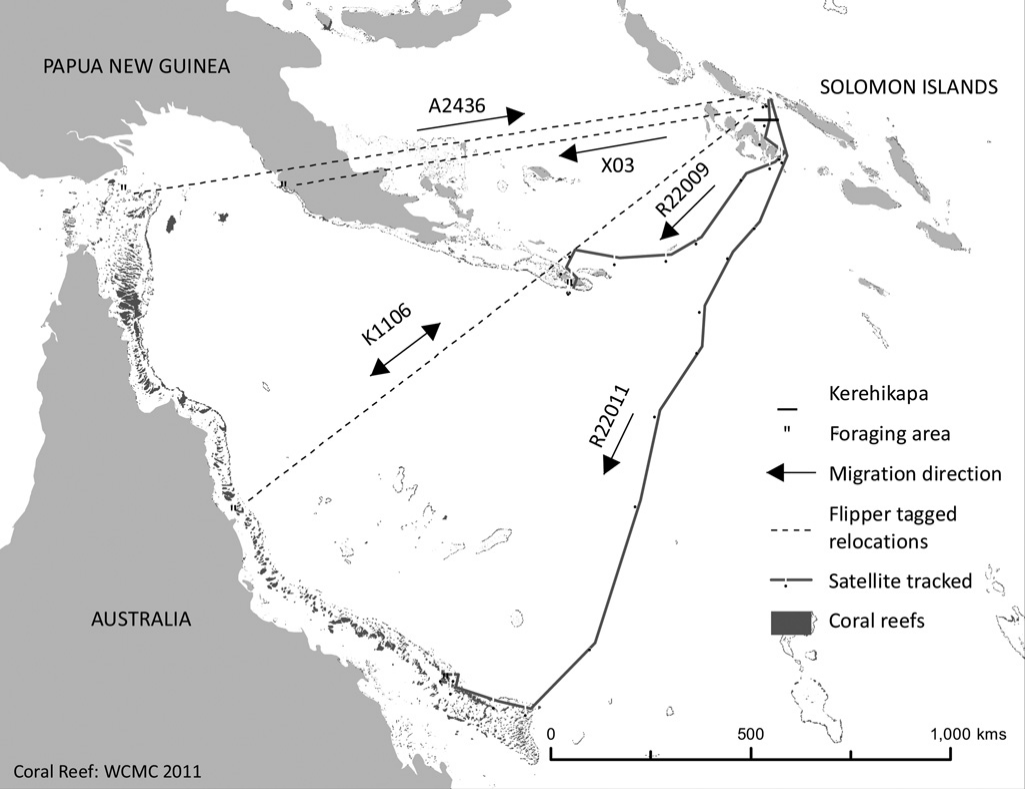
Migrations of adult female hawksbill turtles to and from Kerehikapa beach, Arnavon Islands. Data are derived from three flipper tag recoveries (---); (A2436, [26]), (X03, Vaughan and Spring [25]) and this study (K11060). The migrations of two female turtles that were fitted with telemetry tags (——) on Kerehikapa beach in July 2001 (R22009) and August 2001 (R22011) are also shown.

The movements of five female hawksbills between their foraging grounds and the Kerehikapa nesting beach are shown in Fig. 2. The earliest record was an adult female that was tagged at Kerehikapa in December 1976 (Tag number: X03) and killed on its foraging grounds at Fisherman’s Island, Central Province, Papua New Guinea in February 1979 [25]. The second record was a female (Tag number: A2436) that was first tagged on its foraging grounds at Sakeman Reef, Torres Strait in March 1979, and was observed laying at Kerehikapa in February 1980 [26]. The third record was a female turtle (Tag number: K11060) that was captured six times between 1998 and 2011. The female was first captured as an immature foraging female on Coombe Reef, northern GBR in 1998 and 1999, and in 2005 and 2008 as a foraging adult [27]. She was also recorded nesting on Kerehikapa in June 2004 and May 2011. Also shown is information from two nesting female turtles that were deployed with satellite transmitters in July and August 2001 [13]. The first of these turtles (Tag number: R22009) was tagged at Kerehikapa in July 2001, and by mid-September 2001 it had travelled back to its foraging grounds at Tagula Island in eastern Papua New Guinea. The second turtle (Tag number: R22011) was tagged in August 2001, and by late October 2001 it had travelled back to its foraging grounds in the southern end of the GBR of Australia. The minimum linear distances travelled by these five turtles ranged from 800–1650 kilometres.

### Yearly estimates of laying activity

**Fig 3:**
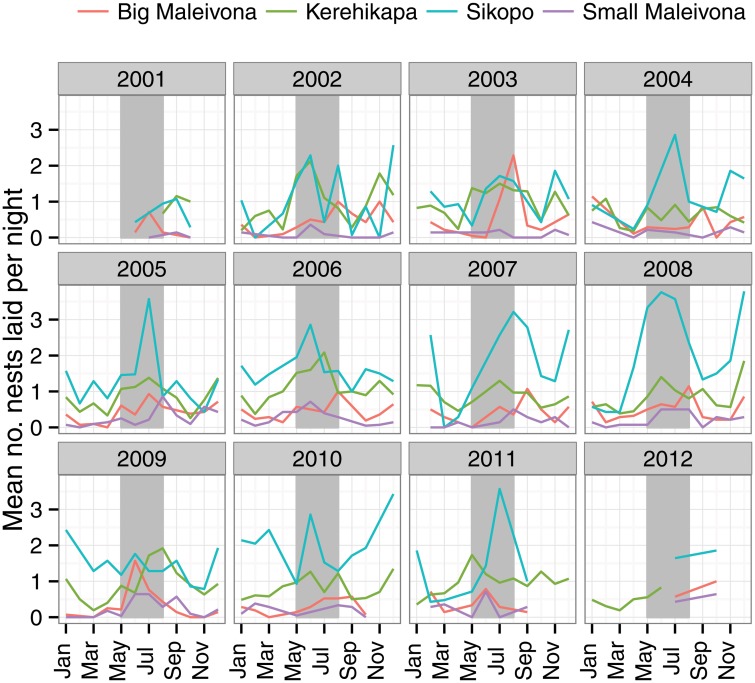
The seasonality of nesting hawksbill turtles in Arnavons. The grey vertical bars show the May-August period.

Hawksbill turtles nest throughout the year on the Arnavons, with peak nesting activity occurring approximately May—July each year during the austral winter (Fig. 3). In some years a second distinct peak also occurs in December-January. In some years (2008, 2009 and 2010), this secondary peak was higher than the May-July peak.

**Fig 4:**
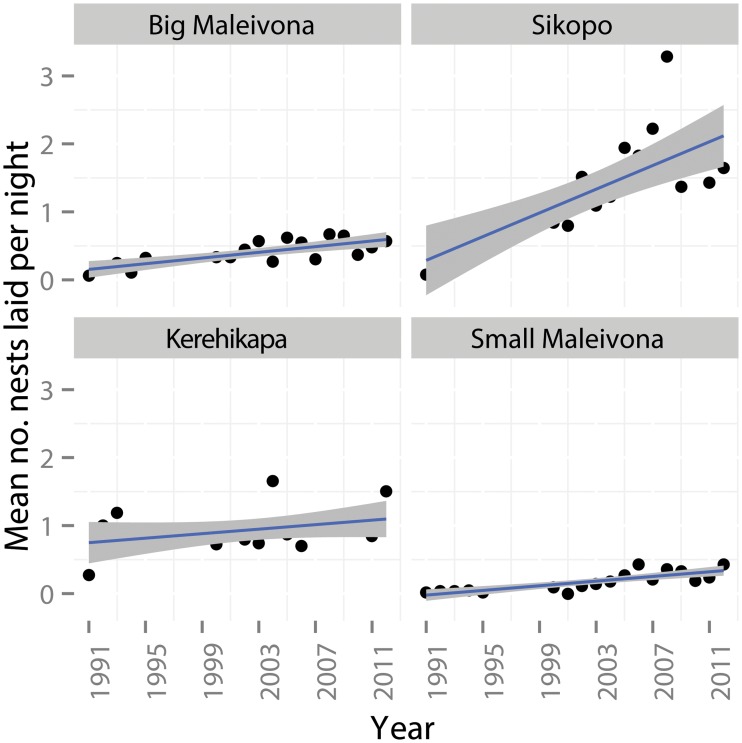
Average number of nests sighted per night at Big Maleivona, Kerehikapa, Sikopo and Small Maleivona during the peak nesting period between 1991 and 2012. Grey bars show 95% confidence intervals around the regression line.

Over the 22 year period there was a significant increasing trend in the number of nests laid per night (Fig. 4, Table 1). Model selection via AIC showed that the model estimating separate rates of increase for each beach provided the best fit (AIC = 87, vs AIC = 91 for the next best model), suggesting a significant increase in number of nests observed over time at some beaches and little change at others. Since the surveys started there has been an overall increase of ~0.42 [0, 0.84] nests per night on Kerehikapa and Big Maleivona, and an increase of ~1.35 [0.75, 2.1], at Sikopo, while Small Maleivona showed no significant increase.

**Table 1:** Parameter estimates from a linear model estimating the effect of time and beach on the mean number of nests sighted per night. The model includes interactions between beach and time, which are shown with the symbol ":". In parameterizing the model, the reference beach used was Big Maleivona.

	Estimate	Std. Error	t value	Pr(>|t|)
(Intercept)	0.1327	0.1532	0.87	0.3897
yr	0.0209	0.0109	1.92	0.0592
IslandKerehikapa	0.5997	0.2181	2.75	0.0078
IslandSikopo	0.0667	0.2167	0.31	0.7591
IslandSmall Maleivona	-0.1716	0.2167	-0.79	0.4314
yr:IslandKerehikapa	-0.0044	0.0154	-0.28	0.7777
yr:IslandSikopo	0.0663	0.0154	4.30	0.0001
yr:IslandSmall Maleivona	-0.0038	0.0154	-0.25	0.8056

**Fig 5:**
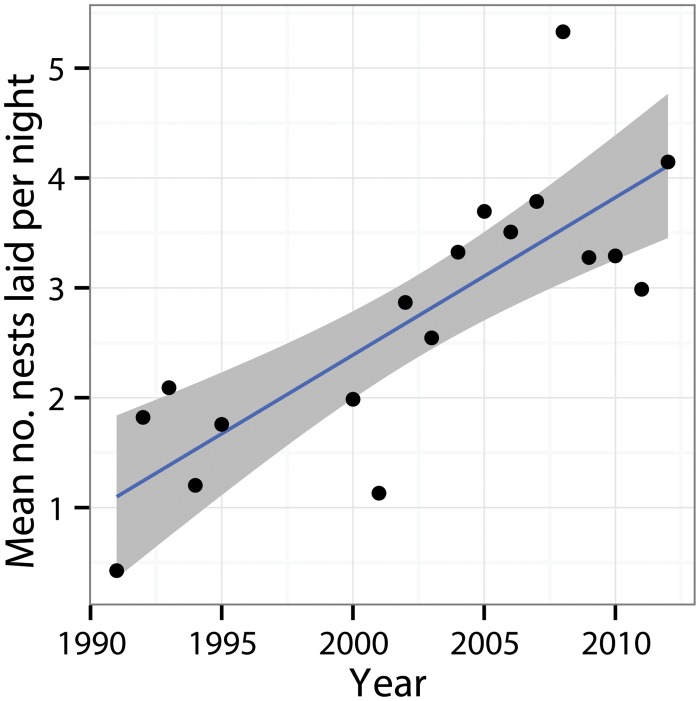
Trends in the average number of hawksbill nests laid per night in the Arnavons group during the peak season (May-August) between 1991–2012. Grey bars show 95% confidence intervals around the regression line.

The mean number of hawksbill clutches laid per night during the peak nesting period for the entire Arnavons group is shown in Fig. 5. The mean number of nest laid per night during the 2008–2012 period (3.85, SE 0.42) was more than twice that recorded during the peak period in 1991–1995 (1.44, SE 0.29, t test, P = 0.01). A linear model through these points suggests an increase of 0.14 clutches per night every year, or one additional clutch per night every seven years.

### Remigration rates and remigration intervals

**Fig 6:**
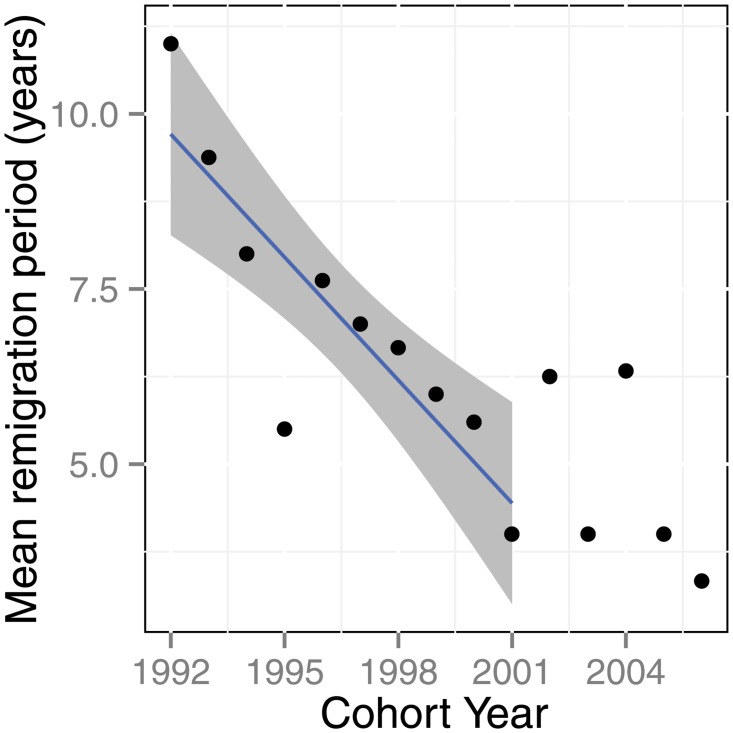
Mean remigration period for cohorts of turtles in the years between 1991 and 2007. Blue line indicates predicted values for a poisson regression of migration period against years since 1991. Grey area indicates 95% confidence intervals around the regression line. The slop of the regression line indicates a reduction in the remigration interval by 0.5/yr.

The mean proportion of turtles from the pre protection era (1991–1995) that remigrated within a 12 year window was 0.067 (SE = 0.027). The mean proportion of turtles in the post protection era (1996–2000) that remigrated within a 12 year window was twice as high; 0.137 (SE 0.021). The mean remigration interval for the breeding season’s cohorts that were followed for at least 12 years beyond their initial year of tagging is shown in Fig. 6. There were significant differences among the mean remigration intervals for these years cohorts (t-test, P = 0.011). A poisson regression of remigration interval against cohort year showed a significant decline in the average remigration interval by 5.8 [3.9, 7.2] years over the 10 years between 1991–2001 (p = 0.002, Fig. 6). During the 21 years for potential recapture no hawksbills were recorded nesting with a one year remigration interval, two year remigration intervals were rare, but increased in frequency in the later cohorts (Table 2).

**Table 2:** Summary of tagging related data from nesting hawksbills tagged at Kerehikapa and subsequent remigration recaptures at Kerehikapa.

Breeding seasons at Kerehikapa Island																							
Year		91	92	93	94	95	96	97	98	99	0	1	2	3	4	5	6	7	8	9	10	11	12
Management status		No Protection					Protection																
Total turtles		11	27	41	34	34	64	36	14	33	44	58	42	38	30	62	75	57	69	70	48	45	15
New turtles tagged		11	27	41	34	34	64	35	14	32	39	50	39	31	24	50	64	49	62	55	38	40	12
Remigrants		0	0	0	0	0	0	1	0	1	5	8	3	7	6	12	11	8	7	15	10	5	3
% new taggings		100	100	100	100	100	100	97.2	100	97.0	88.6	86.2	92.9	81.6	80.0	80.6	85.3	86.0	89.9	78.6	79.2	88.9	80.0
**Year First seen**	1991	**Breeding season**																					
	1992																						
	1993																						
	1994																						
	1995																						
	1996																						
	1997			1																			
	1998																						
	1999			1																			
	2000		1	1		1	1	1															
	2001			2		2	3			1													
	2002			1	1		1																
	2003		2				1	1	1		1	1											
	2004						1	2		1	1	1											
	2005			1			3	1	1	3	2	1		1									
	2006		1			1	2		1	1	1	2	1		1								
	2007			1	1			1		1		1		2		1							
	2008			1								3	2				1						
	2009		1		1	1	1	2		1	2	2		2	1	1							
	2010							2		3	1		1			2	1						
	2011								1				1		2	1							
	2012																2		1				

## Discussion

The aim of this work was to draw on 22 years of monitoring data from the Arnavon Islands turtle nesting beaches in order to understand hawksbill population dynamics, and to use that information to evaluate various conservation efforts. These data proved effective in reconstructing long-term patterns in migration, nesting and rates of return. We use these results below to provide an overview of population mobility and seasonality. We then turn to the issue of conservation and demonstrate that the conservation efforts are effective. These results are significant in that they provide the first known evidence of recovery for a western pacific hawksbill rookery.

### Population mobility and seasonality

Our findings build upon earlier research which has shown that Solomon Islands hawksbill turtles are a shared resource, with many of the hawksbills that nest in Solomon Islands having migrated from their foraging grounds in Australia, Torre Straits and Papua New Guinea [31]. The five migrating turtles reported on here travelled 800–1650 kilometres between their foraging areas and nesting beaches, far greater distances than those reported for hawksbills in the Hawaiian Islands, where nine satellite tracked females travelled distances of 90 to 345 km between their nesting and foraging grounds [32]. Studies from the Caribbean have also revealed that the majority of hawksbills travel less than 500 km between nesting and foraging grounds, although some females migrate distances of over 1500 km [33, 34].

One of the turtles demonstrated strong site fidelity for both her foraging reef and nesting beach, being captured four times at her foraging reef on the northern GBR and two times at Kerehikapa nesting beach over a 14 year period. This finding confirms that a proportion of the turtles that nest at ACMCA come from a foraging population on the GBR that is highly protected yet in decline [35]. It is conceivable that declines in foraging populations of hawksbill turtles on the GBR [35] relate in part to the high mortality that these turtles experience when nesting at unprotected beaches in Melanesia.

At the Arnavon Islands hawksbill turtles nest throughout the year, with a peak nesting period occurring in the austral winter. A less pronounced rise in nesting activity occurs in December-January, though this peak sometimes overshadows nesting activity in the winter period. This seasonality differs from many other rookeries in the region. In Queensland, Australia and Papua New Guinea peak hawksbill nesting occurs in the austral summer [4, 36].

### The efficacy of conservation efforts

The success of the conservation effort in Arnavon Islands was evident in four areas; an increase in annual laying activity, increased remigration rates, shortened remigration intervals and an increasing proportion of remigrants in the annual Arnavons cohort.

#### Increased annual laying activity and remigration rates

Following the national ban on commercial hunting in late 1993 and the establishment of the ACMCA in late 1995 the number of nests laid in the ACMCA has more than doubled. Given that female hawksbill turtles do not mature until 31–36 years of age [4], increasing nest numbers are not due to an increasing number of hatchlings surviving in the post protection period, maturing and returning to nest at the ACMCA. Instead the rise in the number of nests appears to be driven by two factors. Firstly, after 1995 nesting hawksbills had a greater chance of surviving to lay all of their clutches within a nesting season, and secondly, a greater proportion of females are surviving to return in a subsequent nesting season. Our tagging study provides evidence of this increased survival, with the average remigration rate for turtles tagged after the establishment of the ACMCA (1996–2000) being double that of the pre protection (1991–1995) period. Rapid increases in hawksbill nest numbers have also been documented in the Seychelles and the Caribbean after harvest levels were reduced and strictly controlled [37–40], with some depleted hawksbill populations in these regions now considered stable and recovered [34].

#### Shortened remigration intervals

The declining mean remigration interval in the post protection period and the fact that the shortest remigration interval of two years was only observed in cohorts sampled after 1995 provides further evidence of population recovery. It is conceivable that turtles that nested and survived in the early 1990s and then remigrated two years later died before being resighted at Kerehikapa. Whereas those turtles that nested after 1995 and then remigrated two years later had a greater chance of survival. Ramohia [23] reports heavy hunting pressure at the Arnavons in 1991 and none of the turtles tagged in 1991 were ever resighted, suggesting they may have all been killed at the Arnavons or in their nearby inter-nesting habitat that year.

#### Increased proportion of remigrants

Broderick [18] studied the subsistence harvest of turtles taken from reefs adjacent to the ACMCA boundaries (the Arnavon turtles inter nesting habitat) in the mid-1990s. He calculated that less than 10% of the adult female hawksbills he examined were remigrants, indicative of a turtle population that had experienced immense hunting pressure [18]. Results from our tagging survey show that by 2003 the number of remigrants had increased to around 20%. This increase indicates a significant improvement, but is still suggestive of a turtle population subject to heavy hunting pressure. In nesting hawksbill populations where mortality is low, the vast majority of females nesting in any one season are remigrants [4].

### Threats to the ACMCA hawksbill population

Solomon Islands has one of the highest domestic consumption of turtles in the world [1], with Broderick [18] estimating that in the 1990s fishers from Kia, Wagina and Katupika communities harvested 845 hawksbills turtles annually for subsistence purposes. Similar levels continue to this day (JP personal observations), and with rapid population growth in Choiseul and Isabel Provinces [41], rising levels of harvesting from the inter nesting habitat pose a significant threat to the viability of the ACMCA. Compounding this issue is the varying levels of poaching that are known to have occurred inside the ACMCA since it was established [28, 42].

The illegal trade in turtles for consumption (primarily shipped to the capital Honiara) is also common and there is a black market for hawksbill turtle shell in Solomon Islands, with some shell sold to Asian buyers in Honiara [42] and some sold to local carvers for the production of jewellery. Although sale of turtle products is banned under CITES this ban is not enforced in Solomon Islands. For example, hawksbill shell earrings and bangles can currently be purchased in the departure lounge of the Honiara international airport. A similar scenario occurs in Papua New Guinea [36].

## Conclusion

By the early 1990s the Arnavons hawksbill population had experienced 150 years of commercial exploitation and population numbers were at an all-time low. Today, two decades after a national ban on exporting turtles and the establishment of the ACMCA, the Arnavons hawksbill populations is showing encouraging signs of recovery. The number of nests laid at the ACMCA and the remigration rate has doubled following the establishment of the ACMCA. It is noteworthy that while the current number of turtle nests being laid per night is twice as high as the early 1990s, they may be only a fraction of what they were in the 1960s, with McKeown [11] reporting that over two nights in 1963 one hunting party captured 20 hawksbill turtles from Sikopo beach.

Furthermore, given that commercial exploitation of the Arnavons rookery commenced in the 1840s [9], it is likely that by the 1960s the Arnavons hawksbill population had already been in decline for over a century [13]. Such shifting baselines [43] underscore the importance of utilising the disciplines of anthropology, archaeology and history when attempting to understand rates of human induced change [44].

Finally, we acknowledge that the future of the Arnavons hawksbill turtle population is far from secure. Subsistence harvesting in the inter nesting areas, poaching and the illegal trade in this species all threaten its viability. But despite all these challenges, the ACMCA is an inspiring example of how changes in policy, inclusive community-based management and long term commitment are helping to turn the tide for one of the most charismatic and endangered species on our planet.

## Supporting Information

S1 Table Number of daily beach counts conducted at Kerehikapa by month from 1991–2012. (DOCX)

S2 Table Number of weekly beach counts conducted at Sikopo, Big Maleivona and Small Maleivona. (DOCX)

S1 Text Analytical considerations with the CMR data. (DOCX)
