# Supplementary material for: Solomon Islands Largest Hawksbill Turtle Rookery Shows Signs of Recovery after 150 Years of Excessive Exploitation
Source: PLoS One. 2015 Apr 8;10(4):e0121435. doi: 10.1371/journal.pone.0121435 (PMC4390367; doi:10.1371/journal.pone.0121435)
Supplement: S1 Table — (DOCX) [file pone.0121435.s001.docx]

| **Year** | **Jan** | **Feb** | **Mar** | **Apr** | **May** | **Jun** | **Jul** | **Aug** | **Sep** | **Oct** | **Nov** | **Dec** | **Total (year)** |
| --- | --- | --- | --- | --- | --- | --- | --- | --- | --- | --- | --- | --- | --- |
| 1991 | - | - | - | - | 1 | 30 | 31 | 4 | - | - | - | - | **66** |
| 1992 | - | - | - | - | - | 26 | 31 | 27 | - | - | - | - | **84** |
| 1993 | - | - | - | - | 5 | 30 | 31 | 24 | - | - | - | - | **90** |
| 1994 | - | - | - | - | - | 28 | 31 | 25 | - | - | - | - | **84** |
| 1995 | - | - | - | - | - | 12 | 31 | 31 | 1 | - | - | - | **75** |
| 1996 | - | - | - | - | - | - | - | - | - | - | - | - | **-** |
| 1997 | - | - | - | - | - | - | - | - | - | - | - | - | **-** |
| 1998 | - | - | - | - | - | - | - | - | - | - | - | - | **-** |
| 1999 | - | - | - | - | - | - | - | - | - | - | - | - | **-** |
| 2000 | - | - | - | - | - | 29 | 31 | 27 | - | - | - | - | **87** |
| 2001 | - | - | - | - | - | - | - | 18 | 13 | 15 | - | - | **46** |
| 2002 | 15 | 5 | 8 | 22 | 18 | 8 | 19 | 11 | 25 | 8 | 23 | 11 | **173** |
| 2003 | 17 | 9 | 16 | 25 | 24 | 17 | 28 | 19 | 28 | 21 | 22 | 21 | **247** |
| 2004 | 28 | 25 | 30 | 22 | 30 | 29 | 22 | 18 | 15 | 19 | 15 | 12 | **265** |
| 2005 | 25 | 23 | 24 | 24 | 28 | 24 | 29 | 25 | 29 | 27 | 25 | 24 | **307** |
| 2006 | 26 | 24 | 25 | 23 | 29 | 25 | 24 | 25 | 22 | 29 | 24 | 24 | **300** |
| 2007 | 23 | 19 | 27 | 26 | 31 | 29 | 27 | 31 | 29 | 31 | 28 | 29 | **330** |
| 2008 | 30 | 28 | 31 | 29 | 28 | 30 | 31 | 31 | 30 | 31 | 30 | 20 | **349** |
| 2009 | 31 | 28 | 31 | 30 | 24 | 28 | 25 | 24 | 26 | 30 | 30 | 27 | **334** |
| 2010 | 31 | 28 | 31 | 29 | 31 | 30 | 30 | 27 | 26 | 30 | 27 | 26 | **346** |
| 2011 | 31 | 27 | 24 | 28 | 26 | 30 | 26 | 26 | 30 | 26 | 28 | 28 | **330** |
| 2012 | 31 | 26 | 26 | 28 | 27 | 29 | - | - | - | - | - | - | **167** |
| **Total (month)** | **288** | **242** | **273** | **286** | **302** | **434** | **447** | **393** | **274** | **267** | **252** | **222** |  |
| **Total** | **3680** | | | | | | | | | | | | |
