# Supplementary material for: Solomon Islands Largest Hawksbill Turtle Rookery Shows Signs of Recovery after 150 Years of Excessive Exploitation
Source: PLoS One. 2015 Apr 8;10(4):e0121435. doi: 10.1371/journal.pone.0121435 (PMC4390367; doi:10.1371/journal.pone.0121435)
Supplement: S2 Table — (DOCX) [file pone.0121435.s002.docx]

| **Year** | **Island** | **Jan** | **Feb** | **Mar** | **Apr** | **May** | **Jun** | **Jul** | **Aug** | **Sep** | **Oct** | **Nov** | **Dec** |
| --- | --- | --- | --- | --- | --- | --- | --- | --- | --- | --- | --- | --- | --- |
| 1991 | Sikapo | - | - | - | - | - | 4 | 4 | 1 | - | - | - | - |
| 1992 | Sikapo | - | - | - | - | - | 4 | 4 | 4 | - | - | - | - |
| 1993 | Sikapo | - | - | - | - | 1 | 4 | 4 | 4 | - | - | - | - |
| 1994 | Sikapo | - | - | - | - | - | 4 | 4 | 4 | - | - | - | - |
| 1995 | Sikapo | - | - | - | - | - | 2 | 4 | 4 | 1 | - | - | - |
| 2000 | Sikapo | - | - | - | - | - | 4 | 4 | 4 | - | - | - | - |
| 2001 | Sikapo | - | - | - | - | - | 2 | - | 4 | 2 | 1 | - | - |
| 2002 | Sikapo | 3 | 2 | - | 3 | 3 | 2 | 2 | 1 | 2 | 1 | 2 | 1 |
| 2003 | Sikapo | - | 1 | 2 | 2 | 3 | 2 | 2 | 1 | 3 | 2 | 2 | 2 |
| 2004 | Sikapo | 2 | - | - | 3 | 2 | - | 1 | 3 | 1 | 1 | 1 | 2 |
| 2005 | Sikapo | 2 | 3 | 3 | 3 | 4 | 3 | 3 | 1 | 4 | 3 | 1 | 3 |
| 2006 | Sikapo | 2 | 3 | 3 | 1 | 2 | 1 | 4 | 1 | 1 | 3 | 2 | 2 |
| 2007 | Sikapo | - | 1 | 1 | 1 | 3 | - | 4 | 1 | 2 | 2 | 2 | 1 |
| 2008 | Sikapo | 1 | 1 | 1 | 3 | 3 | 3 | 1 | 2 | 3 | 3 | 2 | 2 |
| 2009 | Sikapo | 2 | - | 1 | 4 | 4 | 2 | 3 | 2 | 2 | 3 | 1 | 2 |
| 2010 | Sikapo | 2 | 3 | 1 | - | 2 | 3 | 3 | 3 | 1 | 2 | - | 1 |
| 2011 | Sikapo | 1 | 1 | 3 | - | 3 | 2 | 1 | - | 1 | - | - | - |
| 2012 | Sikapo | - | - | - | - | - |  | 2 | - | - | 2 | - | - |
| 1991 | Big Maleivona | - | - | - | - | - | 4 | 4 | 1 | - | - | - | - |
| 1992 | Big Maleivona | - | - | - | - | - | 4 | 4 | 4 | - | - | - | - |
| 1993 | Big Maleivona | - | - | - | - | 1 | 4 | 4 | 4 | - | - | - | - |
| 1994 | Big Maleivona | - | - | - | - | - | 4 | 4 | 4 | - | - | - | - |
| 1995 | Big Maleivona | - | - | - | - | - | 2 | 4 | 4 | 1 | - | - | - |
| 2000 | Big Maleivona | - | - | - | - | - | 4 | 4 | 4 | - | - | - | - |
| 2001 | Big Maleivona | - | - | - | - | - | 1 | 1 | 1 | 2 | 2 | - | - |
| 2002 | Big Maleivona | 2 | 1 | - | 3 | 4 | 2 | 3 | 1 | 3 | 1 | 2 | 1 |
| 2003 | Big Maleivona | - | 1 | 2 | 2 | 3 | 2 | 2 | 1 | 3 | 2 | 2 | 2 |
| 2004 | Big Maleivona | 1 | - | - | 2 | 2 | - | 2 | 2 | 1 | 1 | 1 | 1 |
| 2005 | Big Maleivona | 2 | 2 | 3 | 3 | 4 | 2 | 2 | 1 | 3 | 3 | 1 | 3 |
| 2006 | Big Maleivona | 2 | 3 | 1 | 1 | 2 | - | 4 | 1 | - | 3 | 2 | 2 |
| 2007 | Big Maleivona | - | 2 | 1 | 2 | 3 | - | 3 | 2 | 2 | 2 | 1 | 1 |
| 2008 | Big Maleivona | 1 | 1 | 2 | 3 | 2 | 2 | 1 | 1 | 2 | 2 | 2 | 1 |
| 2009 | Big Maleivona | 2 | - | 1 | 4 | 4 | 2 | 3 | 2 | 1 | 3 | 2 | 2 |
| 2010 | Big Maleivona | 3 | 3 | 1 | - | 3 | 3 | 3 | 3 | 1 | 2 | - | - |
| 2011 | Big Maleivona | - | 1 | 2 | - | 3 | 2 | 1 | - | 1 | - | - | - |
| 2012 | Big Maleivona | - | - | - | - | - | - | 2 | - | - | 2 | - | - |
| 1991 | Small Maleivona | - | - | - | - | - | 4 | 4 | 1 | - | - | - | - |
| 1992 | Small Maleivona | - | - | - | - | - | 4 | 4 | 4 | - | - | - | - |
| 1993 | Small Maleivona | - | - | - | - | 1 | 4 | 4 | 4 | - | - | - | - |
| 1994 | Small Maleivona | - | - | - | - | - | 4 | 4 | 4 | - | - | - | - |
| 1995 | Small Maleivona | - | - | - | - | - | 2 | 4 | 4 | 1 | - | - | - |
| 2000 | Small Maleivona | - | - | - | - | - | 4 | 4 | 4 | - | - | - | - |
| 2001 | Small Maleivona | - | - | - | - | - | - | 1 | - | 2 | 1 | - | - |
| 2002 | Small Maleivona | 2 | - | - | 2 | 4 | 2 | 3 | - | 3 | 1 | 2 | 1 |
| 2003 | Small Maleivona | - | 1 | 2 | 1 | 3 | 1 | 2 | 1 | 3 | 2 | 2 | 2 |
| 2004 | Small Maleivona | 1 | - | - | 2 | 2 | - | 2 | - | 1 | - | 1 | 2 |
| 2005 | Small Maleivona | 2 | 2 | 3 | 3 | 4 | 2 | 2 | 1 | 3 | 3 | 1 | 3 |
| 2006 | Small Maleivona | 1 | 3 | 1 | 1 | 2 | 1 | 4 | 1 | - | 3 | 2 | 2 |
| 2007 | Small Maleivona | - | 2 | 1 | 2 | 2 | - | 3 | 2 | 2 | 2 | 1 | 1 |
| 2008 | Small Maleivona | 1 | 1 | 2 | 2 | 2 | 2 | - | 1 | 1 | 2 | 2 | 1 |
| 2009 | Small Maleivona | 1 | - | 1 | 4 | 4 | 2 | 2 | 2 | 1 | 3 | 2 | 2 |
| 2010 | Small Maleivona | 3 | 3 | 1 | - | 3 | 3 | 3 | 3 | 1 | 2 | - | - |
| 2011 | Small Maleivona | - | 1 | 2 | - | 3 | 2 | 1 | - | 1 | - | - | - |
| 2012 | Small Maleivona | - | - | - | - | - | - | 2 | - | - | 2 | - | - |
| **Total (month)** | | **39** | **42** | **41** | **57** | **91** | **117** | **150** | **108** | **62** | **67** | **39** | **43** |
| **Grand total** | | **856** | | | | | | | | | | | |
