# Supplementary material for: Solomon Islands Largest Hawksbill Turtle Rookery Shows Signs of Recovery after 150 Years of Excessive Exploitation
Source: PLoS One. 2015 Apr 8;10(4):e0121435. doi: 10.1371/journal.pone.0121435 (PMC4390367; doi:10.1371/journal.pone.0121435)
Supplement: S1 Text — (DOCX) [file pone.0121435.s003.docx]

**S1 Text. Analytical considerations with the CMR data.**

This paper represents the first empirical attempt to pull together all available monitoring data from the ACMCA. While the linear models presented here are robust and provide meaningful evidence of population recovery, a more definitive analysis using capture mark recapture histories for variation in survivorship would provide better evidence of increasing population numbers and survival rates. Unfortunately, such an analysis is not feasible given the present data constraints.

When preparing this paper we used several existing analytical approaches in an attempt to estimate survival rates, including program MARK and WinBUGS.  In both cases, we found that survival rates were grossly over-estimated and capture rates underestimated, both signatures of bias due to temporary migration. We elaborate here on why this is the case and why including results from these analyses would be misleading.

Because turtles migrate away from the nesting beaches between years, they are only available for capture during years in which they are nesting. This situation represents a case of temporary, non-random migration from the study area.  In cases where migration is at random (ie all turtles have the same probability of being outside the sampling area), estimates of survival need not be biased.  However in this case, turtles that have recently left the study area are less likely to nest than turtles that have been away for some time. As such, migration is non-random and will bias both estimated survival and capture rates. We have confirmed this finding with extensive simulation studies.

Several options are available for dealing with this sort of temporary migration in a CMR framework, all of which require additional data. One option is the robust design sampling framework, which makes use of multiple captures within a short time period to more accurately estimate detection rates.  We investigated the use of robust design analyses in this case, recognizing that turtles return multiple times within a period of months and are often captured multiple times within this period.  However as the number of times that each turtle returns is unknown and variable, it is impossible to use the multiple recaptures to better estimate recapture rates or the remigration period.

A further option to account for the remigration period is to explicitly include it as a parameter to be estimated in the model. However, because capture rates and migration rates are confounded, we have no ability to separate the two. Further Bayesian simulation studies showed that even with a highly informative prior distribution for the remigration period, estimates of survival were significantly biased.
